# Narrative review of ferroptosis in obesity

**DOI:** 10.1111/jcmm.17701

**Published:** 2023-03-05

**Authors:** Lian‐Ping He, Zi‐Xuan Zhou, Cui‐Ping Li

**Affiliations:** ^1^ School of Medicine Taizhou University Zhejiang China

**Keywords:** ferroptosis, iron metabolism, obesity, review

## Abstract

Obesity is widely recognized as a major global health problem caused by a chronic energy imbalance resulting from a combination of excess caloric intake and insufficient energy expenditure. Excessive energy intake and physical inactivity are traditional risk factors for obesity. Obesity is a risk factor for many diseases, including hypertension, diabetes and tumours. Recent studies have found a strong link between ferroptosis and obesity. Ferroptosis is an iron‐dependent regulated cell death caused by iron overload and reactive oxygen species‐dependent excessive accumulation of lipid peroxidation. Ferroptosis is involved in many biological processes, such as amino acid metabolism, iron metabolism and lipid metabolism. Some potential strategies to reduce the adverse effects of ferroptosis on obesity are suggested and future research priorities are highlighted.

## 1 INTRODUCTION

Obesity is a serious, chronic, complex and relapsing disease associated with changes in adipose tissue mass, distribution or function, which is associated with severe morbidity and increased mortality. Obesity has become the world's largest chronic disease. According to the World Health Organization (WHO) 2017 Global Disease Report, an estimated 107.7 million children and 603.7 million adults were obese globally in 2015. The overall obesity rate is 5.0% and 12.0%, respectively, which is the country with the highest obesity rate in China.^1^

Obesity is caused by the interaction of genetic, environmental, physiological, behavioural and sociocultural factors.^2^ In general, obesity is caused by multiple factors that affect energy intake and expenditure. The main route of energy intake is through food intake or feeding behaviour. Feeding behaviour is regulated by the hunger‐satiety circuit in the central nervous system. The arcuate nucleus (ARC) of the hypothalamus is thought to be the primary site for integrating hunger‐satiety circuit. ARC within the hypothalamus produces orexinergic neuropeptides agouti‐related peptide (AgRP)/neuropeptide Y (NPY), proapipimelanocortin (POMC)/cocaine‐and amphetamine‐regulated transcript (CART). Stimulation of AgRP/NPY neurons leads to increased food intake and weight gain.^3^ However, lateral cells of the ARC release the POMC‐derived peptide alpha‐melanocyte‐stimulating hormone (α‐MSH) potently reduce food intake.^4^

In addition to the central nervous system, energy intake and expenditure are also influenced by lifestyle. For example, there is a physiological link between sleep and energy balance.^5^ Lack of sleep is a risk factor for overeating and weight gain, and molecules such as orexin and insulin play an important role in controlling sleep and energy intake. Lifestyle can also induce activation or inhibition of adenosine monophosphate‐activated protein kinase/mammalian target of rapamycin (AMPK/mTOR) signalling pathway, which regulates the energy balance.^6^

**FIGURE 1:**
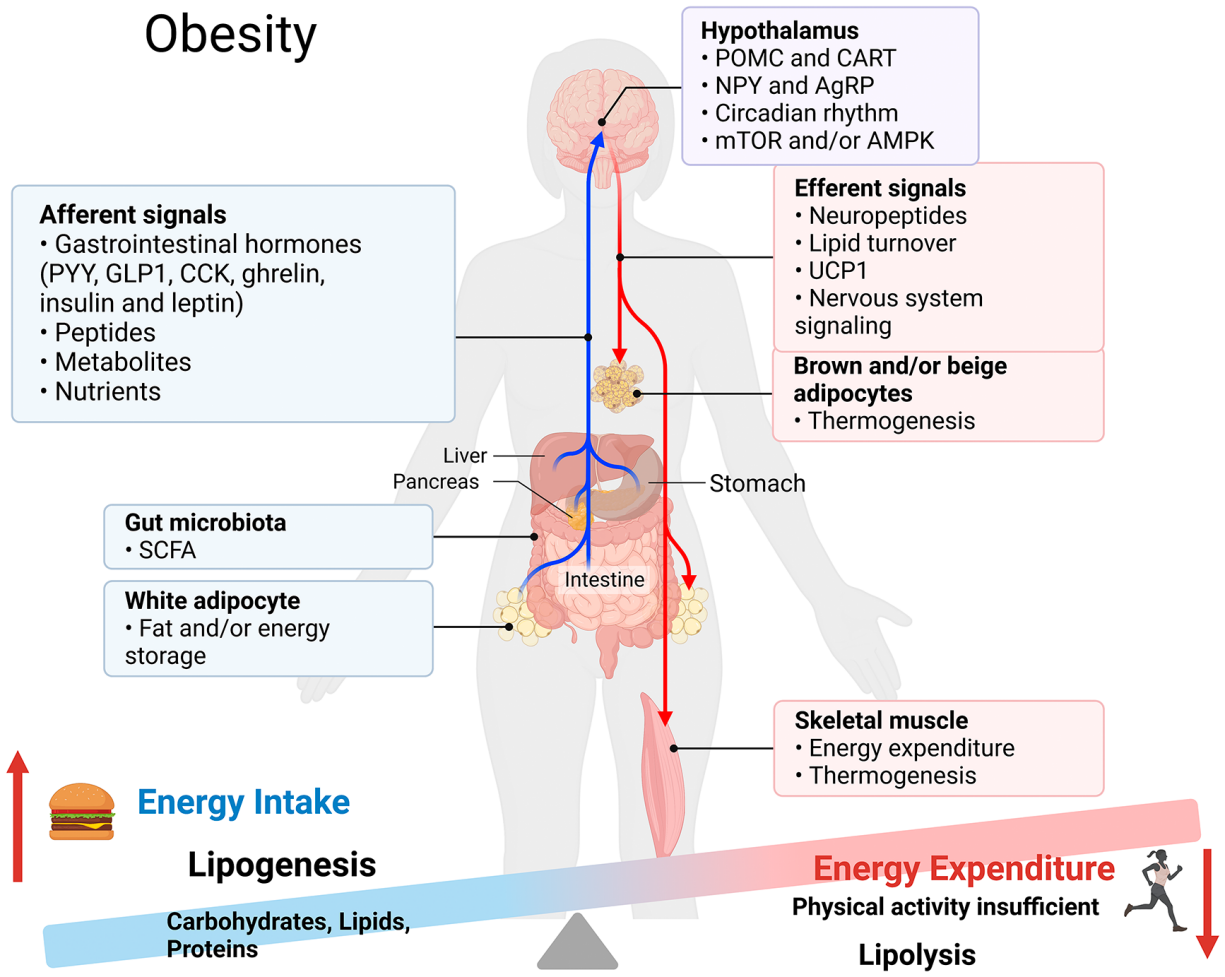
Energy imbalance and obesity.

Thermogenesis is also a way of regulating energy balance. Brown adipose tissue (BAT) is rich in mitochondria containing uncoupling protein 1 (UCP1), which uses energy through thermogenesis.^7^ Shiverless thermogenesis in skeletal muscle is also an attractive strategy to combat obesity.^8^ The gut microbiota also affects host energy balance by regulating genes related to fat absorption and storage. Study found that noni fruit phenolic extract suppresses obesity by increasing short‐chain fatty acid (SCFA)‐producing bacteria and suppressing abnormalities in the gut microbiota.^9^ In addition, glucagon‐like peptide‐1(GLP‐1), incretins and pleiotrophins have pharmacotherapeutic potential in the treatment of obesity.^10^ See Figure 1 for a brief summary of metabolic imbalance and obesity.

**FIGURE 2:**
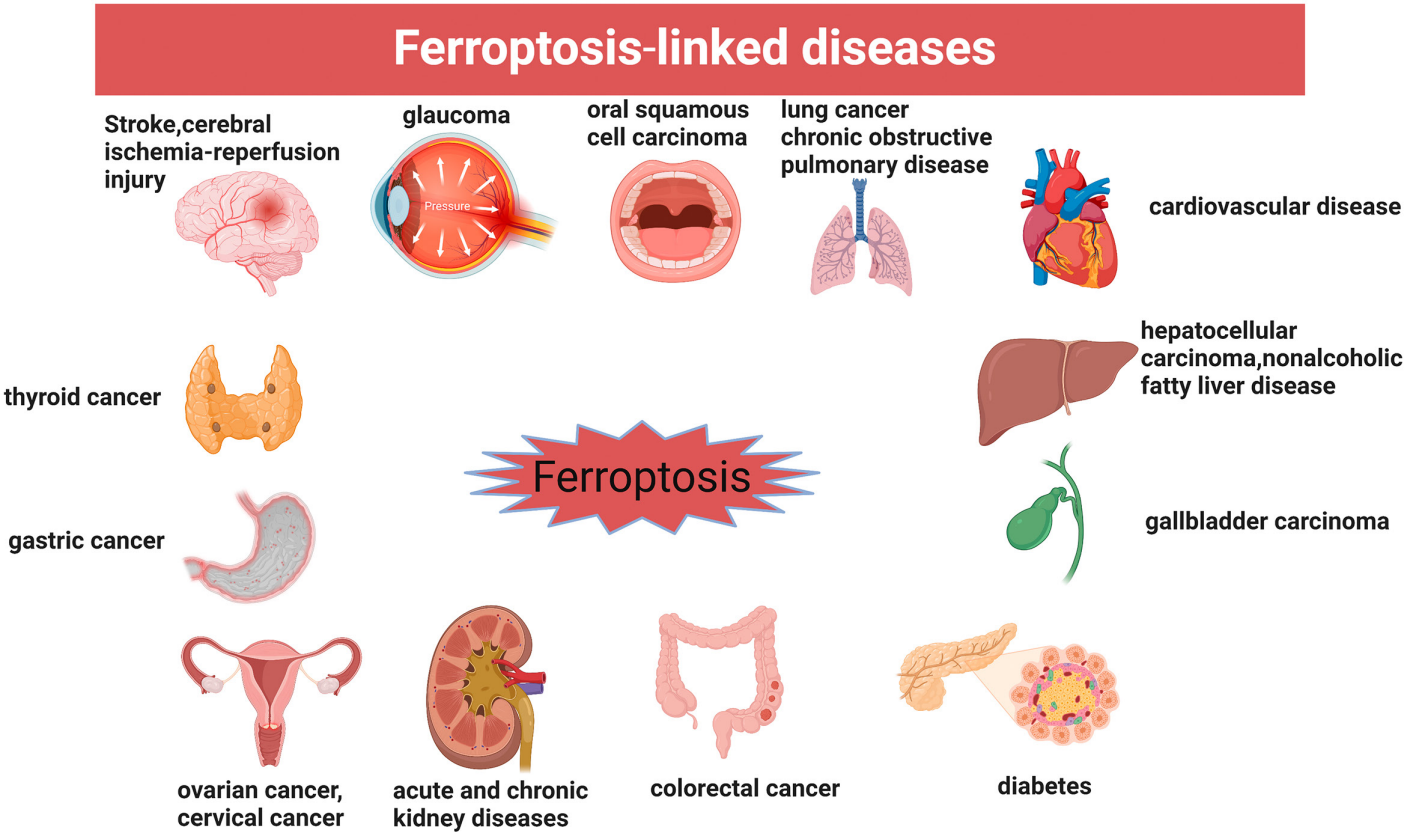
Ferroptosis‐linked diseases.

Ferroptosis is an iron‐dependent regulated cell death (RCD) caused by iron overload and reactive oxygen species (ROS)‐dependent excessive accumulation of lipid peroxidation, first proposed by Dixon et al.^11^ in 2012. Ferroptosis is characterized by changes in mitochondrial morphology, including mitochondrial shrinkage, reduced or absent mitochondrial cristae, and increased mitochondrial membrane density.^12^ Its bioenergetic features are mainly iron accumulation and lipid peroxidation.^12^ It has been established that ferroptosis is involved in the initiation and progression of many diseases. These diseases (Figure 2) included stroke,^13^ colorectal cancer,^14^ hepatocellular carcinoma,^15^ glaucoma,^16^ oral squamous cell carcinoma,^17^ traumatic brain injury,^18^ acute and chronic kidney diseases,^19^ cerebral ischemia–reperfusion injury,^20^ spinal cord injury,^18^ lung cancer,^21^ gastric cancer,^22^ gallbladder carcinoma,^23^ cardiovascular disease,^24^ thyroid cancer,^25^ chronic obstructive pulmonary disease,^26^ ovarian cancer,^27^ cervical cancer,^28^ diabetes^29^, ^30^ and nonalcoholic fatty liver disease.^31^

**FIGURE 3:**
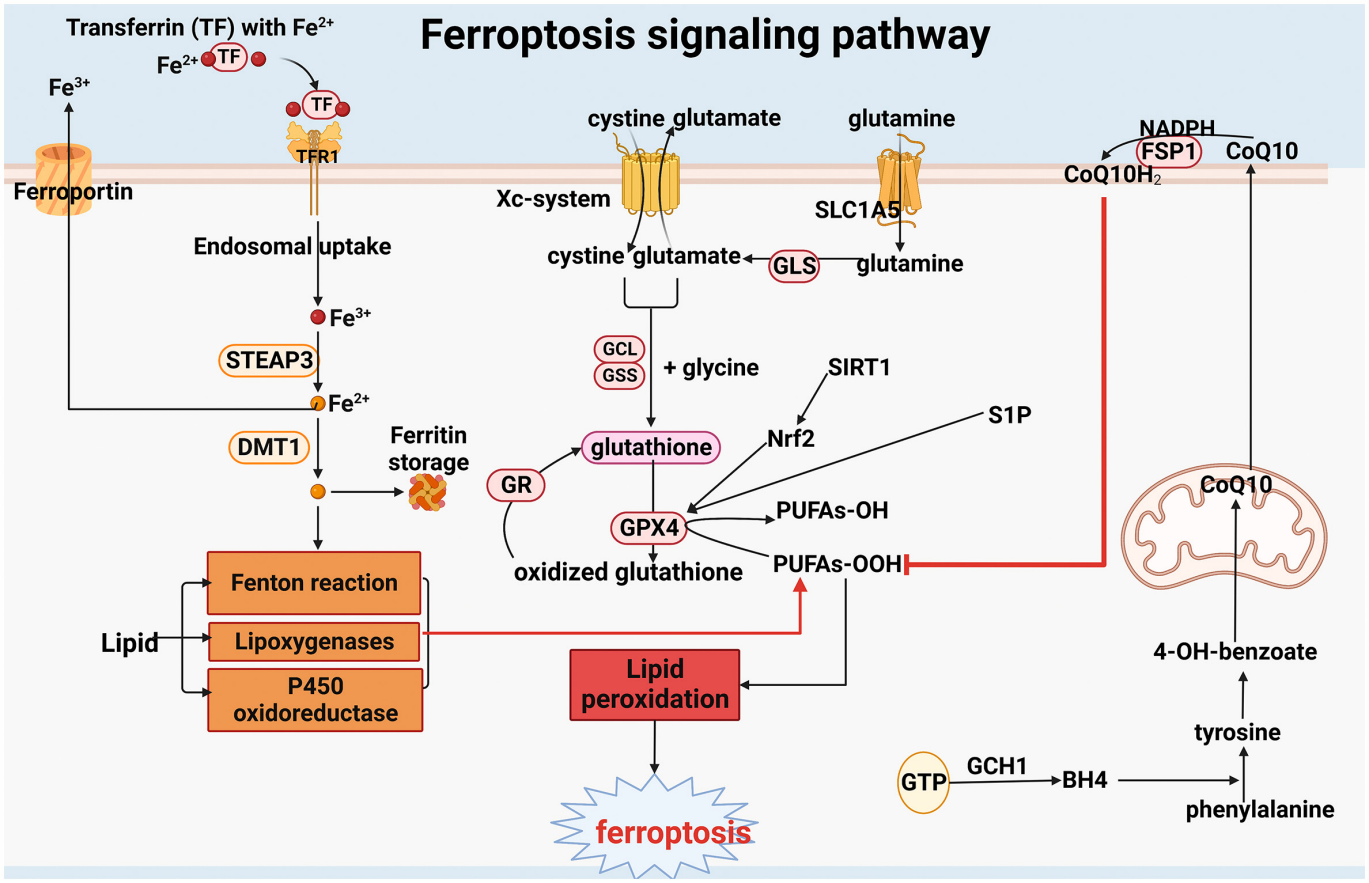
Ferroptosis signalling.

Furthermore, ferroptosis is closely related to many disease processes, such as oxidative stress,^32^ inflammatory response^33^ and autophagy.^34^ In particular, ferroptosis is involved in the pathological process of obesity.^35^ Excessive nutrient intake and fat storage in white adipose tissue (WAT) ultimately leads to obesity. Insufficient iron intake is also linked to overweight or obesity in children and adolescents.^36^ A systematic meta‐analysis of 21 studies (including 13,393 overweight participants and 26,621 non‐obese participants) found a significant association between obesity and iron deficiency.^37^ Therefore, understanding the regulatory pathways of ferroptosis is of great significance for the prevention and treatment of obesity. Ferroptosis is involved in many biological processes, such as amino acid metabolism, iron metabolism, lipid metabolism (Figure 3).

## 2 REGULATORY PATHWAYS OF FERROPTOSIS

### 2.1 The role of iron metabolism in ferroptosis

Iron homeostasis is maintained by a complex network of absorption, transport, utilization, storage and excretion. Transferrin receptor (TFR) is the main mediator of iron entry into cells. Extracellular iron (Fe^3+^) first binds to transferrin (TF), and Fe^3+^‐loaded TF has a high affinity for the extracellular domain of TFR1.^38^ After binding to TFR1, the TF/TFR1 complex is taken up by endocytosis. Due to the acidic environment of the endosome, Fe3^+^ is released from TF and immediately reduced to Fe^2+^ by prostate iron reductase 6 transmembrane epithelial antigen (STEAP3). Release of Fe^2+^ from endosomes to the cytosol via divalent metal transporter 1 (DMT1).^39^ The released Fe^2+^ forms a cytoplasmic labile iron store (LIP), in which iron is metabolically activated. Excess iron is normally stored in ferritin to aid iron detoxification. Ferritin is a natural iron‐storage protein. There are two types of ferritin, heavy chain ferritin (HFn) and light chain ferritin (L‐ferritin, LFn).^40^ Most of the released Fe^2+^ is ultimately used in various physiological processes such as the Fenton reaction. For an overview of the current findings in the role of iron metabolism in ferroptosis, please refer to the other review article.^41^

### 2.2 The lipid metabolism in ferroptosis

**FIGURE 4:**
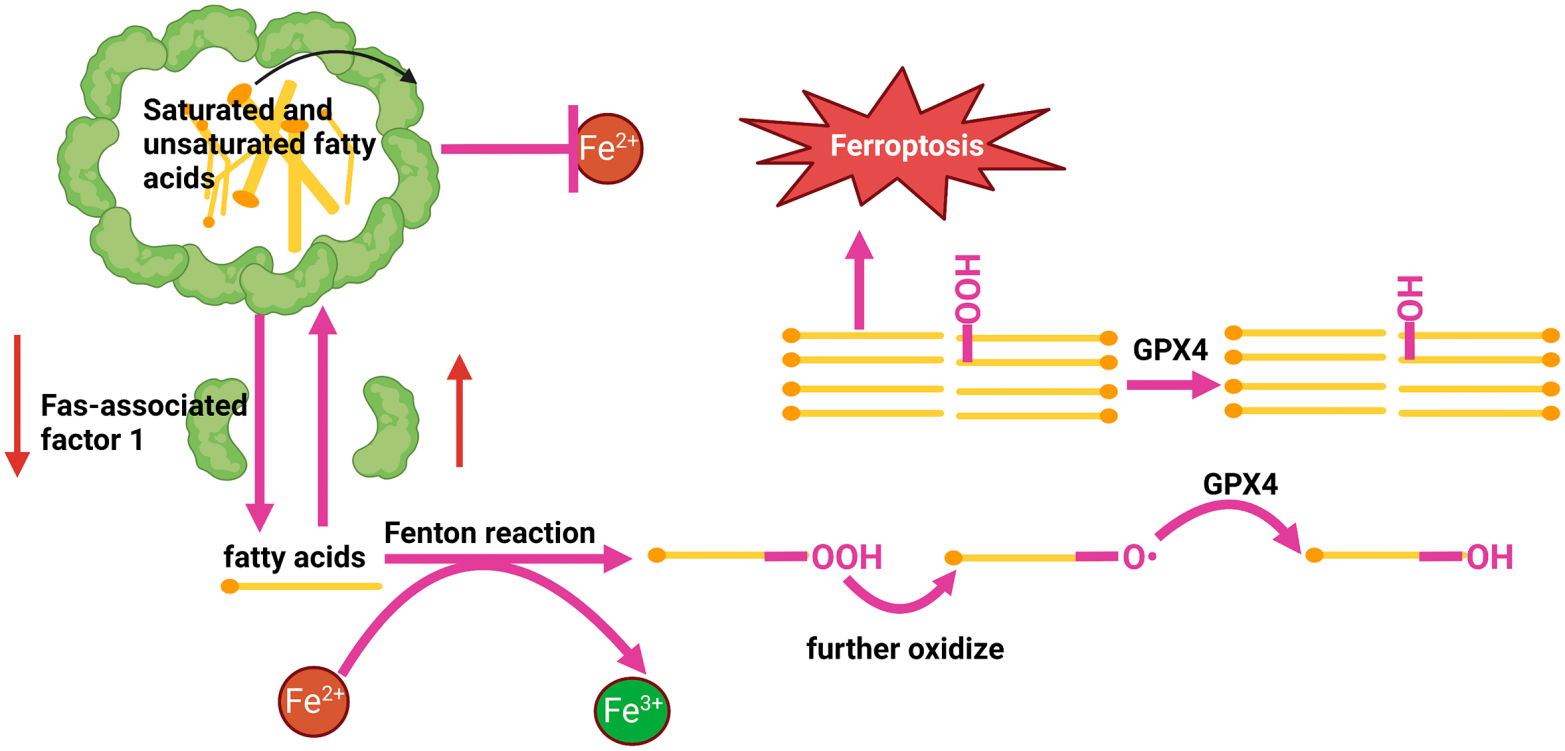
The lipid metabolism in ferroptosis.

It is known that when mammals cannot use carbohydrates to generate adenosine triphosphate (ATP), glucose is mostly converted into fatty acids (lipogenesis) for synthesis and storage of triglyceride (TGs) in the liver and white adipose tissue. Lipid droplets are universal storage organelles for neutral lipids (TGs) that can be found in most cells. Dynamics and functions of lipid droplets have been recently reviewed.^42^ Lipid droplets showed that they were encapsulated by a factor called Fas‐associated factor 1, which protected them from contact with Fe^2+^.^43^ TGs in lipid droplets are hydrolyzed sequentially through three ester bonds to produce glycerol and free fatty acid (FFA). In the cytoplasm, FFA triggers the Fenton reaction under Fe^2+^ overload conditions, and it is easy to generate PUFAs‐OOH. In the presence of Fe^2+^, PUFAs‐OOH is easy to further oxidize to PUFAs‐O^•^, which can seize H from the adjacent PUFAs, start a new round of lipid oxidation reaction, cause a large amount of oxidation of PUFAs, and damage the integrity of the biofilm, eventually leading to cell death. Glutathione peroxidase 4 (GPX4) converts reduced PUFAs‐O^•^ to oxidized PUFAs‐OH and reduces cytotoxic lipid peroxides.^44^, ^45^ Palmitic acid (PA) is the most abundant type of saturated fatty acid in the plasma.^46^ Reduction of lipid synthesis often causes FFA overload which induce mitochondria reactive oxygen species (ROS) generation and endoplasmic reticulum (ER) stress. ROS, including superoxide anion radicals (O_2_^‐•^) and hydrogen peroxide (H_2_O_2_), are continuously produced intracellularly as by‐products of energy metabolism in various types of hepatocytes.^47^ FFAs stimulate nicotinamide adenosine dinucleotide phosphate oxidase (NOXs) to generate more ROS, which also leads to ferroptosis through lipid peroxidation (See Figure 4).

### 2.3 The role of GSH/GPX4 pathway in ferroptosis

Glutathione (GSH) is a potent antioxidant that plays an important role in ferroptosis due to its ability to scavenge excess ROS. Cytoplasmic glutathione is the product of glutamic acid, cysteine and glycine under the catalysis of glutamate cysteine ligase (GCL) and glutathione synthase (GSS). Translocation of glycine from the extracellular to the cytoplasm requires the membrane cystine/glutamate antiporter(Xc‐system). The Xc‐system primarily imports extracellular cystine in exchange for intracellular glutamate.^48^ ROS‐induced ferroptosis can be regulated indirectly by modulating the expression of the Xc‐system and affecting intracellular cystine levels. Increased activity of the Xc‐system promotes the synthesis of GSH, resulting in elevated GSH levels. Expression of the Xc‐system is regulated by the redox‐sensitive transcription factor, nuclear factor‐related Red‐2 factor 2 (Nrf2). Therefor, compounds capable of activating the Nrf2‐antioxidant response element (Nrf2‐ARE) pathway, termed “Nrf2” activators; have received increasing attention for their potential as GSH enhancers. Glutathione peroxidase 4 (GPX4) converts reduced GSH to oxidized glutathione (GSSG) and reduces cytotoxic lipid peroxides.^44^, ^45^ All molecules involved in the regulation of the GSH/GPX4 axis can be potential targets for the regulation of ferroptosis.

### 2.4 CoQ10/FSP1 antioxidant pathway in ferroptosis

The–CoQ10–NAD(P)H/FSP1 pathway is a parallel independent system that, together with GPX4 and GSH, inhibits phospholipid peroxidation and ferroptosis. Ferroptosis suppressor protein 1 (FSP1) was formerly known as mitochondrial apoptosis‐inducing factor 2 (AIFM2). It was initially annotated as a p53‐responsive gene, and later reports showed that it is highly homologous to apoptosis‐inducing factor (AIF).^49^, ^50^, ^51^ FSP1 is a member of the type II NADH: quinone oxidoreductase (NDH‐2) family. So, its intrinsic role is to reduce CoQ10 using NADH.^52^ Two important reports demonstrated the role of FSP1 as a novel suppressor of ferroptosis.^53^, ^54^ N‐myristoylation of FSP1 mediates the recruitment of this protein to lipid droplets and plasma membranes. It is necessary and sufficient to confer resistance to ferroptosis. FSP1 is a membrane‐recruited oxidoreductase that catalyses the transport of reduced NADH analogs of coenzyme Q10 into the lipid bilayer, thereby inhibiting lipid peroxidation. The ferroptosis inhibitor iFSP1 acts mainly by directly inhibiting FSP1. FIN56 can bind and activate squalene.^55^ As an antioxidant pathway parallel to GSH/GPX4 pathway, CoQ10/FSP1 antioxidant pathway must also play an important role in ferroptosis.

### 2.5 GCH1‐BH4‐phospholipid pathway in ferroptosis

The GCH1‐BH4‐phospholipid pathway also has an antioxidant effect on ferroptosis via CoQ10. Tetrahydrobiopterin (BH4) is a redox‐active cofactor for several key enzymes involved in the production of nitric oxide, neurotransmitters and aromatic amino acids^56^, ^57^ Guanosine triphosphate cyclohydrolase 1 (GCH1) is the rate‐limiting enzyme of BH4 biosynthesis. BH4 may promote the synthesis of CoQ10 by converting phenylalanine into tyrosine that can be further converted to 4‐OH‐benzoate, a precursor of CoQ10. Besides, BH4 can directly protect two‐tailed PUFA phosphatidylcholines from peroxidation to resist ferroptosis. This pathway is also a GPX4 ‐independent pathway **(**Figure 3 **).**

## 3 POTENTIAL STRATEGIES TO IMPROVE FERROPTOSIS

### 3.1 Regulating iron metabolism in ferroptosis

Since iron is tightly regulated by controlling iron uptake, storage, utilization and efflux. Therefore, regulation of iron metabolic processes can inhibit ferroptosis. For example, it can regulate iron absorption. Calcium is a noncompetitive inhibitor of iron importer divalent metal transporter 1 (DMT1) on the intestinal iron absorption. Vitamin D is known to promote calcium absorption. Study found that micronutrient (Vitamin D) deficiencies are common in obesity.^58^ Obesity is a risk factor for diabetes, and people with diabetes may also be deficient in vitamin D.^59^ Interventions consisting of calcium and iron may provide new ideas for obesity prevention.

### 3.2 Regulating GPX4 pathway in ferroptosis

Modulating GPX4 activity and expression is also a potential strategy to alleviate ferroptosis. Silent information regulator factor 2‐related enzyme 1 (SIRT1)/Nrf2/GPX4 signalling pathway as a potential therapeutic target for inhibiting ferroptosis.^60^ SIRT1 is a nicotinamide adenine dinucleotide‐dependent histone deacetylase, promote longevity and protect individual organisms from age‐associated diseases.^61^ In additional, sphingosine 1‐phosphate (S1P) alleviates radiation‐induced ferroptosis in ovarian granulosa cells by upregulating GPX4. Physical activity activates the SIRT1 pathway,^62^ which has also been linked to creativity.^63^ Physical activity can also indirectly regulate the activity and expression of GPX4. As mentioned above, GPX4 signalling pathway as a potential therapeutic target for inhibiting ferroptosis. Therefore, we speculate that the S1P signalling pathway may be an indirect regulatory target of ferroptosis. The effects of sphingolipid metabolism disorders on endothelial cells have been reviewed in detail elsewhere.^64^

## 4 CONCLUSIONS AND FUTURE PERSPECTIVES

Ferroptosis is a newly defined type of RCD driven by the accumulation of iron‐dependent lipid ROS and associated with the development of many diseases. Ferroptosis participates in the occurrence of obesity, and seriously threatens the health of all population. Common mechanisms of ferroptosis in obesity include iron overload, lipid peroxide hyperplasia, GPX4 inhibition and systemic Xc‐inhibition. Calcium is a noncompetitive inhibitor of ferrous transporter 1 (DMT1) during intestinal iron absorption. Calcium also induces the downregulation of genes related to iron absorption. It is speculated that vitamin D supplementation may inhibit iron absorption by promoting calcium absorption, and ultimately inhibit the occurrence of ferroptosis. There are few studies on the role of iron‐calcium connection in obesity. Therefore, whether iron‐calcium connection plays an important role in iron overload‐induced obesity needs further investigation. Modulating GPX4 activity and expression is also a potential strategy to alleviate ferroptosis. Silent information regulator factor 2‐related enzyme 1 (SIRT1)/Nrf2/GPX4 signalling pathway as a potential therapeutic target for inhibiting ferroptosis. S1P attenuates radiation‐induced ferroptosis in ovarian granulosa cells through upregulation of GPX4. Therefore, we speculate that the S1P signalling pathway may be an indirect regulatory target of ferroptosis.

## AUTHOR CONTRIBUTIONS

**Lian‐Ping He:** Conceptualization (equal); writing – original draft (lead); writing – review and editing (lead). **Zi‐Xuan Zhou:** Conceptualization (equal); writing – original draft (equal). **Cui‐Ping Li:** Writing – original draft (equal); writing – review and editing (lead).

## CONFLICT OF INTEREST STATEMENT

The author confirms that there are no conflicts of interest.

## Data Availability

Data sharing not applicable to this article as no datasets were generated or analysed during the current study.

## References

[1] Ezzati M , Bentham J , Di Cesare M , et al. Worldwide trends in body‐mass index, underweight, overweight, and obesity from 1975 to 2016: a pooled analysis of 2416 population‐based measurement studies in 128.9 million children, adolescents, and adults. Lancet. 2017;390(10113):2627‐2642. https://doi.org10.1016/S0140‐6736(17)32129‐3 29029897 10.1016/S0140-6736(17)32129-3 PMC5735219

[2] Bray GA , Fruhbeck G , Ryan DH , Wilding JP . Management of obesity. Lancet. 2016;387(10031):1947‐1956. 10.1016/S0140-6736(16)00271-3 26868660

[3] Krashes MJ , Koda S , Ye C , et al. Rapid, reversible activation of AgRP neurons drives feeding behavior in mice. J Clin Invest. 2011;121(4):1424‐1428. 10.1172/JCI46229 21364278 PMC3069789

[4] Yoon NA , Jin S , Kim JD , et al. UCP2‐dependent redox sensing in POMC neurons regulates feeding. Cell Rep. 2022;41(13):111894. 10.1016/j.celrep.2022.111894 36577374 PMC9885759

[5] Chaput JP , McHill AW , Cox RC , et al. The role of insufficient sleep and circadian misalignment in obesity. Nat Rev Endocrinol. 2023;19(2):82‐97. 10.1038/s41574-022-00747-7 36280789 PMC9590398

[6] Li Y , Cheng Y , Zhou Y , et al. High fat diet‐induced obesity leads to depressive and anxiety‐like behaviors in mice via AMPK/mTOR‐mediated autophagy. Exp Neurol. 2022;348:113949. 10.1016/j.expneurol.2021.113949 34902357

[7] Da Eira D , Jani S , Ceddia RB . An obesogenic diet impairs uncoupled substrate oxidation and promotes whitening of the brown adipose tissue in rats. J Physiol. 2023;601(1):69‐82. 10.1113/JP283721 36419345

[8] Li H , Wang C , Li L , Li L . Skeletal muscle non‐shivering thermogenesis as an attractive strategy to combat obesity. Life Sci. 2021;269:119024. 10.1016/j.lfs.2021.119024 33450257

[9] Wang R , Wang L , Wang S , et al. Phenolics from noni (Morinda citrifolia L.) fruit alleviate obesity in high fat diet‐fed mice via modulating the gut microbiota and mitigating intestinal damage. Food Chem. 2023;402:134232. 10.1016/j.foodchem.2022.134232 36137374

[10] Holst JJ , Jepsen SL , Modvig I . GLP‐1—incretin and pleiotropic hormone with pharmacotherapy potential. Increasing secretion of endogenous GLP‐1 for diabetes and obesity therapy. Curr Opin Pharmacol. 2022;63:102189. 10.1016/j.coph.2022.102189 35231672

[11] Dixon SJ , Lemberg KM , Lamprecht MR , et al. Ferroptosis: an iron‐dependent form of nonapoptotic cell death. Cell. 2012;149(5):1060‐1072. 10.1016/j.cell.2012.03.042 22632970 PMC3367386

[12] Li J , Kang R , Tang D . Monitoring autophagy‐dependent ferroptosis. Methods Cell Biol. 2021;165:163‐176. 10.1016/bs.mcb.2020.10.012 34311865

[13] Xu Y , Li K , Zhao Y , Zhou L , Liu Y , Zhao J . Role of ferroptosis in stroke. Cell Mol Neurobiol. 2023;43(1):205‐222. 10.1007/s10571-022-01196-6 35102454 PMC11415219

[14] Yang L , Zhang Y , Zhang Y , Fan Z . Mechanism and application of ferroptosis in colorectal cancer. Biomed Pharmacother. 2023;158:114102. 10.1016/j.biopha.2022.114102 36528917

[15] Huang Z , Xia H , Cui Y , Yam JWP , Xu Y . Ferroptosis: from basic research to clinical therapeutics in hepatocellular carcinoma. J Clin Transl Hepatol. 2023;11(1):207‐218. 10.14218/JCTH.2022.00255 36406319 PMC9647096

[16] Yang M , So KF , Lam WC , Yin Lo AC . Ferroptosis and glaucoma: implications in retinal ganglion cell damage and optic nerve survival. Neural Regen Res. 2023;18(3):545‐546. 10.4103/1673-5374.350196 36018170 PMC9727424

[17] Fan X , Zhong Y , Yuan F , Zhang L , Cai Y , Liao L . A ferroptosis‐related prognostic model with excellent clinical performance based on the exploration of the mechanism of oral squamous cell carcinoma progression. Sci Rep. 2023;13(1):1461. 10.1038/s41598-023-27676-3 36702843 PMC9880000

[18] Li QS , Jia YJ . Ferroptosis: a critical player and potential therapeutic target in traumatic brain injury and spinal cord injury. Neural Regen Res. 2023;18(3):506‐512. 10.4103/1673-5374.350187 36018155 PMC9727428

[19] Zhou Y , Zhang J , Guan Q , Tao X , Wang J , Li W . The role of ferroptosis in the development of acute and chronic kidney diseases. J Cell Physiol. 2022;237(12):4412‐4427. 10.1002/jcp.30901 36260516

[20] Wang Z , Li Y , Ye Y , et al. NLRP3 inflammasome deficiency attenuates cerebral ischemia‐reperfusion injury by inhibiting ferroptosis. Brain Res Bull. 2023;193:37‐46. 10.1016/j.brainresbull.2022.11.016 36435361

[21] Zhang Y , Dong P , Liu N , Yang JY , Wang HM , Geng Q . TRIM6 reduces ferroptosis and chemosensitivity by targeting SLC1A5 in lung cancer. Oxid Med Cell Longev. 2023;2023:9808100. 10.1155/2023/9808100 36654781 PMC9842414

[22] Xu X , Li Y , Wu Y , et al. Increased ATF2 expression predicts poor prognosis and inhibits sorafenib‐induced ferroptosis in gastric cancer. Redox Biol. 2023;59:102564. 10.1016/j.redox.2022.102564 36473315 PMC9723522

[23] Huang HX , Yang G , Yang Y , Yan J , Tang XY , Pan Q . TFAP2A is a novel regulator that modulates ferroptosis in gallbladder carcinoma cells via the Nrf2 signalling axis. Eur Rev Med Pharmacol Sci. 2020;24(9):4745‐4755. 10.26355/eurrev_202005_21163 32432738

[24] Fang X , Ardehali H , Min J , Wang F . The molecular and metabolic landscape of iron and ferroptosis in cardiovascular disease. Nat Rev Cardiol. 2023;20(1):7‐23. 10.1038/s41569-022-00735-4 35788564 PMC9252571

[25] Sekhar KR , Cyr S , Baregamian N . Ferroptosis inducers in thyroid cancer. World J Surg. 2023;47(2):371‐381. 10.1007/s00268-022-06738-z 36195678

[26] Meng D , Zhu C , Jia R , Li Z , Wang W , Song S . The molecular mechanism of ferroptosis and its role in COPD. Front Med (Lausanne). 2022;9:1052540. 10.3389/fmed.2022.1052540 36687445 PMC9852995

[27] Wang CK , Chen TJ , Tan GYT , et al. MEX3A mediates p53 degradation to suppress ferroptosis and facilitate ovarian cancer tumorigenesis. Cancer Res. 2023;83(2):251‐263. 10.1158/0008-5472.CAN-22-1159 36354374 PMC9845988

[28] Jiang M , Song Y , Liu H , Jin Y , Li R , Zhu X . DHODH inhibition exerts synergistic therapeutic effect with cisplatin to induce ferroptosis in cervical cancer through regulating mTOR pathway. Cancers (Basel). 2023;15(2):546. 10.3390/cancers15020546 36672495 PMC9856746

[29] Sha W , Hu F , Xi Y , Chu Y , Bu S . Mechanism of ferroptosis and its role in type 2 diabetes mellitus. J Diabetes Res. 2021;9999612. 10.1155/2021/9999612 34258295 PMC8257355

[30] Luo EF , Li HX , Qin YH , et al. Role of ferroptosis in the process of diabetes‐induced endothelial dysfunction. World J Diabetes. 2021;12(2):124‐137. 10.4239/wjd.v12.i2.124 33594332 PMC7839168

[31] Ji J , Wu L , Wei J , Wu J , Guo C . The gut microbiome and ferroptosis in MAFLD. J Clin Transl Hepatol. 2023;11(1):174‐187. 10.14218/JCTH.2022.00136 36406312 PMC9647110

[32] Wu C , Zhao W , Yu J , Li S , Lin L , Chen X . Induction of ferroptosis and mitochondrial dysfunction by oxidative stress in PC12 cells. Sci Rep. 2018;8(1):574. 10.1038/s41598-017-18935-1 29330409 PMC5766540

[33] Sun Y , Chen P , Zhai B , et al. The emerging role of ferroptosis in inflammation. Biomed Pharmacother. 2020;127:110108. 10.1016/j.biopha.2020.110108 32234642

[34] Zhou B , Liu J , Kang R , Klionsky DJ , Kroemer G , Tang D . Ferroptosis is a type of autophagy‐dependent cell death. Semin Cancer Biol. 2020;66:89‐100. 10.1016/j.semcancer.2019.03.002 30880243

[35] Zhang S , Sun Z , Jiang X , et al. Ferroptosis increases obesity: crosstalk between adipocytes and the neuroimmune system. Front Immunol. 2022;13:1049936. 10.3389/fimmu.2022.1049936 36479119 PMC9720262

[36] Hutchinson C . A review of iron studies in overweight and obese children and adolescents: a double burden in the young? Eur J Nutr. 2016;55(7):2179‐2197. 10.1007/s00394-016-1155-7 26883916

[37] Zhao L , Zhang X , Shen Y , Fang X , Wang Y , Wang F . Obesity and iron deficiency: a quantitative meta‐analysis. Obes Rev. 2015;16(12):1081‐1093. 10.1111/obr.12323 26395622

[38] Miljus G , Malenkovic V , Dukanovic B , Kolundzic N , Nedic O . IGFBP‐3/transferrin/transferrin receptor 1 complexes as principal mediators of IGFBP‐3 delivery to colon cells in non‐cancer and cancer tissues. Exp Mol Pathol. 2015;98(3):431‐438. 10.1016/j.yexmp.2015.03.035 25839091

[39] Yu P , Chang YZ . Brain iron metabolism and regulation. Adv Exp Med Biol. 2019;1173:33‐44. 10.1007/978-981-13-9589-5_3 31456204

[40] Zhou B , Zhang JY , Liu XS , et al. Tom20 senses iron‐activated ROS signaling to promote melanoma cell pyroptosis. Cell Res. 2018;28(12):1171‐1185. 10.1038/s41422-018-0090-y 30287942 PMC6274649

[41] Liu J , Kang R , Tang D . Signaling pathways and defense mechanisms of ferroptosis. FEBS J. 2022;289(22):7038‐7050. 10.1111/febs.16059 34092035

[42] Olzmann JA , Carvalho P . Dynamics and functions of lipid droplets. Nat Rev Mol Cell Biol. 2019;20(3):137‐155. 10.1038/s41580-018-0085-z 30523332 PMC6746329

[43] Cui S , Simmons G Jr , Vale G , et al. FAF1 blocks ferroptosis by inhibiting peroxidation of polyunsaturated fatty acids. Proc Natl Acad Sci U S A. 2022;119(17):e2107189119. 10.1073/pnas.2107189119 35467977 PMC9169925

[44] Ursini F , Maiorino M . Lipid peroxidation and ferroptosis: the role of GSH and GPx4. Free Radic Biol Med. 2020;152:175‐185. 10.1016/j.freeradbiomed.2020.02.027 32165281

[45] Mandal PK , Seiler A , Perisic T , et al. System x(c)‐ and thioredoxin reductase 1 cooperatively rescue glutathione deficiency. J Biol Chem. 2010;285(29):22244‐22253. 10.1074/jbc.M110.121327 20463017 PMC2903358

[46] Abdelmagid SA , Clarke SE , Nielsen DE , et al. Comprehensive profiling of plasma fatty acid concentrations in young healthy Canadian adults. PLoS One. 2015;10(2):e0116195. 10.1371/journal.pone.0116195 25675440 PMC4326172

[47] Masarone M , Rosato V , Dallio M , et al. Role of oxidative stress in pathophysiology of nonalcoholic fatty liver disease. Oxid Med Cell Longev. 2018;2018:9547613. 10.1155/2018/9547613 29991976 PMC6016172

[48] Sato H , Tamba M , Ishii T , Bannai S . Cloning and expression of a plasma membrane cystine/glutamate exchange transporter composed of two distinct proteins. J Biol Chem. 1999;274(17):11455‐11148. 10.1074/jbc.274.17.11455 10206947

[49] Horikoshi N , Cong J , Kley N , Shenk T . Isolation of differentially expressed cDNAs from p53‐dependent apoptotic cells: activation of the human homologue of the drosophila peroxidasin gene. Biochem Biophys Res Commun. 1999;261(3):864‐869. 10.1006/bbrc.1999.1123 10441517

[50] Ohiro Y , Garkavtsev I , Kobayashi S , et al. A novel p53‐inducible apoptogenic gene, PRG3, encodes a homologue of the apoptosis‐inducing factor (AIF). FEBS Lett. 2002;524((1–3)):163‐171. 10.1016/s0014-5793(02)03049-1 12135761

[51] Wu M , Xu LG , Li X , Zhai Z , Shu HB . AMID, an apoptosis‐inducing factor‐homologous mitochondrion‐associated protein, induces caspase‐independent apoptosis. J Biol Chem. 2002;277(28):25617‐25623. 10.1074/jbc.M202285200 11980907

[52] Elguindy MM , Nakamaru‐Ogiso E . Apoptosis‐inducing factor (AIF) and its family member protein, AMID, are rotenone‐sensitive NADH:ubiquinone oxidoreductases (NDH‐2). J Biol Chem. 2015;290(34):20815‐20826. 10.1074/jbc.M115.641498 26063804 PMC4543644

[53] Doll S , Freitas FP , Shah R , et al. FSP1 is a glutathione‐independent ferroptosis suppressor. Nature. 2019;575(7784):693‐698. 10.1038/s41586-019-1707-0 31634899

[54] Bersuker K , Hendricks JM , Li Z , et al. The CoQ oxidoreductase FSP1 acts parallel to GPX4 to inhibit ferroptosis. Nature. 2019;575(7784):688‐692. 10.1038/s41586-019-1705-2 31634900 PMC6883167

[55] Shimada K , Skouta R , Kaplan A , et al. Global survey of cell death mechanisms reveals metabolic regulation of ferroptosis. Nat Chem Biol. 2016;12(7):497‐503. 10.1038/nchembio.2079 27159577 PMC4920070

[56] Cronin SJF , Seehus C , Weidinger A , et al. The metabolite BH4 controls T cell proliferation in autoimmunity and cancer. Nature. 2018;563(7732):564‐568. 10.1038/s41586-018-0701-2 30405245 PMC6438708

[57] Kraft VAN , Bezjian CT , Pfeiffer S , et al. GTP Cyclohydrolase 1/tetrahydrobiopterin counteract ferroptosis through lipid remodeling. ACS Cent Sci. 2020;6(1):41‐53. 10.1021/acscentsci.9b01063 31989025 PMC6978838

[58] Yao Y , Zhu L , He L , et al. A meta‐analysis of the relationship between vitamin D deficiency and obesity. Int J Clin Exp Med. 2015;8(9):14977‐14984. 26628980 PMC4658869

[59] He LP , Song YX , Zhu T , Gu W , Liu CW . Progress in the relationship between vitamin D deficiency and the incidence of type 1 diabetes mellitus in children. J Diabetes Res. 2022;2022:5953562. 10.1155/2022/5953562 36090587 PMC9463035

[60] Li C , Wu Z , Xue H , et al. Ferroptosis contributes to hypoxic‐ischemic brain injury in neonatal rats: role of the SIRT1/Nrf2/GPx4 signaling pathway. CNS Neurosci Ther. 2022;28(12):2268‐2280. 10.1111/cns.13973 36184790 PMC9627393

[61] Baeken MW . Sirtuins and their influence on autophagy. J Cell Biochem. 2023. 10.1002/jcb.30377 [Online ahead of print] 36745668

[62] Wu C , Li X , Zhao H , et al. Resistance exercise promotes the resolution and recanalization of deep venous thrombosis in a mouse model via SIRT1 upregulation. BMC Cardiovasc Disord. 2023;23(1):18. 10.1186/s12872-022-02908-y 36639616 PMC9837998

[63] Li CP , Liu XH , Wang XJ , He LP . Trait creativity, personality, and physical activity: a structural equation model. Ann Palliat Med. 2023;12(1):141‐149. 10.21037/apm-22-1310 36747388

[64] Lai Y , Tian Y , You X , Du J , Huang J . Effects of sphingolipid metabolism disorders on endothelial cells. Lipids Health Dis. 2022;21(1):101. 10.1186/s12944-022-01701-2 36229882 PMC9563846

